# Community-based medication disposal pilot initiative in southwest tribal communities

**DOI:** 10.1186/s40621-021-00360-8

**Published:** 2021-12-08

**Authors:** Isaac Ampadu, Robert Morones, Andrea Tsatoke, Lacie Ampadu, Martin Stephens, William C. Crump, David Bales

**Affiliations:** 1 IHS - Western Arizona District Office, 1553 West Todd Drive, Suite 104, Tempe, AZ 85283 USA; 2 IHS - Phoenix Area Office, 40 North Central Avenue, Suite 600, Phoenix, AZ 85004 USA; 3 IHS - Eastern Arizona District Office, 5448 S. White Mountain Blvd., Suite 220, Lakeside, AZ 85929 USA; 4 3685 E. Perkinsville Street, Gilbert, AZ 85295 USA; 5 IHS - Reno District Office, 1150 Financial Blvd. Suite 500, Reno, NV 89502 USA; 6 IHS - Rhinelander District Office, 9a South Brown St, Rhinelander, WI 54501 USA; 7 IHS - Oklahoma Area Office, 701 Market Dr, Oklahoma City, OK 73114 USA

**Keywords:** Behavior change, Interventions, Public health, Poisoning, Medication disposal, Community, Opioids, Tribal

## Abstract

**Background:**

Misuse and abuse of prescription drugs including opioids has been a driving force behind the drug overdose epidemic plaguing communities across the USA for more than two decades. Medication accumulation in the home environment can contribute to this issue. However, research on proper disposal in rural communities is limited. For this project, an applied public health approach was used to raise awareness and improve prescription drug disposal practices by pilot testing prescription drug disposal systems in participating communities.

**Methods:**

A community-based disposal project was facilitated with assistance from community partners. The project centered on distribution of drug deactivation bags in homes and medication drop boxes at multiple healthcare facilities.

**Results:**

The team distributed 215 drug deactivation bags to 162 community households resulting in destruction of 8011 pills, 8 medicated dermal patches and 777 mL of liquid medication. A total of 4684 pounds of medication were collected and disposed of through healthcare facility drop boxes.

**Conclusion:**

The strategies identified are scalable and easy to replicate to meet any community's needs in reducing potential challenges of medication diversion.

## Background

Misuse and abuse of prescription drugs, including opioids, has been a driving force behind the overdose epidemic plaguing communities across the nation for more than two decades. As the leading cause of injury-related death, drug overdose claimed the lives of more than 70,000 individuals in 2019 (National Institutes of Health 2020). The impact of prescription drug abuse/misuse has been felt across all racial and ethnic groups; however, American Indian/Alaskan Natives (AI/AN) have felt the magnitude of the crisis. In 2018, AI/AN population had the second-highest drug overdose death rate (14.2 deaths/100,000 people) in the USA (Wilson et al. 2020).

Published literature suggests medication left unsecured in the home contributes to an increased risk of intentional medication abuse, theft, diversion by individuals and unintentional poisoning due to ingestion by small children or pets (McCance-Katz 2019).

The US Food and Drug Administration (FDA) provides guidance for proper medication disposal, which includes disposal at drug take-back sites (e.g., retail pharmacies, police departments), flushing in a domestic sewer system if listed on the FDA approved “Flush List,” which includes some medications sought after for their misuse and/or abuse potential, or other types of medications that can be discarded in the domestic trash (Center for Drug Evaluation and Research n.d.).

Due to the remote setting of many tribal communities, literature is limited on barriers and access to proper medication disposal. This often results in stockpiling medications in the home environment or improperly discarding in the trash or toilet. From a previous project survey conducted by the Indian Health Service (IHS) Injury Prevention Program (IPP), only one out of every five individuals who had been prescribed medications disposed of them properly. To address this issue, IHS introduced strategies to improve disposal.

IHS is the principal healthcare provider and health advocate for AI/AN communities, which includes preventative services offered by IPP. IHS consists of 12 geographical areas across the USA; each area supports a unique group of tribes (Service, I. H. 2020). Within IHS Division of Environmental Health Service, IPP is designed to assist tribal communities to reduce injury-related risk factors. As of 2018, injuries were the leading cause of AI/AN death among those ages 1–54 (National Center for Injury Prevention and Control, C. n.d.). Through collaborative efforts, IPP utilized an applied public health approach to raise awareness, provide education and improve prescription drug disposal practices by pilot testing two drug disposal systems.

## Methods

The project’s goal was to determine if the distribution of drug deactivation bags and medication drop boxes would serve as acceptable options for medication disposal in tribal communities. Both methods met US Drug Enforcement Administration (DEA) requirements. Tribal leadership in participating communities and the appropriate IHS Institutional Review Board formally approved the project.

### Baseline assessment

During routine home safety assessments, medications were frequently observed stockpiled and unsecured throughout the residence. Anecdotal comments were received from tribal elders and other community members expressing the need for education and access to proper medication disposal options. In addition, telephone interviews were conducted by IPP staff to document IHS and tribally operated healthcare facility pharmacy department perspectives to better understand community disposal options.

During interviews, the following were asked:

1. Is education provided to the community regarding proper disposal of expired/unused medications?
2. Is there a location within the healthcare facility for community members to dispose of medications?
3. If there is no system in place, would the facility participate in a project focused on safe medication disposal?

Responses indicated that none of the healthcare facilities had an openly accessible system for collecting and disposing of unused or expired medications. Baseline information suggested accessible options for medication disposal would reduce unused/excess prescription medications in the home environment.

### Drug deactivation bags

Drug deactivation bags were provided to IPP at no additional cost as part of a statewide response to the opioid epidemic; however, there is an average cost of $3.90 per bag. Over 1700 bags were received for distribution among tribal communities across Arizona. This initiative was piloted within six communities over a three-month period.

#### Product description

Drug deactivation bags selected for this project contained carbon-activated powder that neutralizes medication. Medium-size bags were distributed, which allow for destruction of 45 pills, six fluid ounces of liquid medication, and six dermal patches. Deactivation bags work by depositing medications, adding warm water, sealing, and shaking the bag to mix contents. After 30 s, contents are deactivated and safe for deposit in the domestic trash.

#### Tools development

An educational flyer was created to promote participation and provide step-by-step instructions of deactivation bags.

A community partner distributed the flyer and bags along with an in-person overview. A data collection form was developed for use during distribution. The forms gathered the following information: patient’s age and quantity of unused/expired medication (whether pills, patches, liquid or other); quantity and medication type being disposed; reason for disposal (i.e., discontinued, expired, unused, or other); and whether medication was prescribed for pain relief. Discontinued medications were classified as: medical provider, original prescriber, instructed individuals to discontinue use; expired medications were those beyond the use-by or expiration date; unused medications were defined as medications individuals elected to stop taking. An individual completing the form could select multiple options as reasons for medication disposal, such as expired and discontinued, or unused and expired. Space was provided for both community partners and community members to initial and verify the destruction of medications. A master data collection spreadsheet was created to track usage for all pilot sites.

#### Distribution

Pharmacists and community partners distributed drug deactivation bags using multiple methods: (1) during community events, (2) during home or provider office visits, and (3) through door-to-door campaigns. Community partners included Community Health Representatives (CHR) Program and Public Health Nurses (PHN) that provide home health services to community members. The CHR/PHN program is a primary healthcare program that provides services geared toward health promotion, disease prevention, and reducing health risks at the community level for elderly or disabled patients. As part of their role, they advocate for public health at the local level and assist community members with medication distribution.

Prior to implementation, a brief kick-off meeting was held with the community partners to discuss the project goals and deliverables. This meeting also served as a training session to review the project materials, to view a 2-min “how-to” video by the manufacturer, and as an opportunity for hands-on training.

### Medication drop boxes

Baseline assessments revealed IHS and tribally operated healthcare facilities did not have drop boxes for community medication disposal. Although they offered periodic take-back events, they were interested in obtaining a box to expand disposal options for their patients. Twenty-six (26) of the 29 participating sites were in rural setting. This was defined using Rural–Urban Communing Area (RUCA) code methodology (Defining rural population 2021). This portion of the project included healthcare facilities in Arizona, Minnesota, Nevada and Oklahoma.

#### Product description

Stainless-steel collection drop boxes with the capacity to hold 18 to 36 gallons were selected for the project. The box secures to the floor or wall and is equipped with two locks on the main door and a one-way medicine drop. Each box included a removable, prepaid, ship-back liner. When full, liners must be sealed and returned to the vendor via common carrier to undergo proper destruction. Boxes for this project were purchased through approved government vendors and cost between $1300 and $1450 per unit. Annual maintenance, which included liner and common carrier fee, ranged from $465 to $675. Drop box site installations were in accordance with Title 21 Code of Federal Regulation Part 1317 Subpart B enforced by DEA. Requirements included: (1) a DEA license, (2) 24-h monitoring and (3) bolted/secured to floor or wall.

#### Tools development

A flyer was developed and provided to points of contact (POC) highlighting the importance of the medication drop box, benefits to the community and the facility, and DEA requirements for collection sites. A centralized tracking spreadsheet was developed to analyze disposal data made available by the manufacturer through an online portal.

#### Distribution

Funding for drop boxes was site-specific. Chief Pharmacists were the facility POCs and were responsible for obtaining funding for the drop box and its maintenance.

## Results

Key findings from this pilot project included the widespread distribution of medication deactivation bags in conjunction with increased public knowledge and awareness of medication drop box locations.

### Drug deactivation bags

In May 2019, the IPP collaborated with five Arizona tribal communities and distributed 215 drug deactivation bags to 162 community households. These bags were used to destroy 8011 pills, 777 mL (mL) of liquid, and eight medicated dermal patches (Table 1).

**Table 1:** Forms of medications disposed of using the deactivation bags

Types of medications disposed	Number of medications disposed
Pills	8011
Patches	8
Liquid	777 mL

**Fig. 1:**
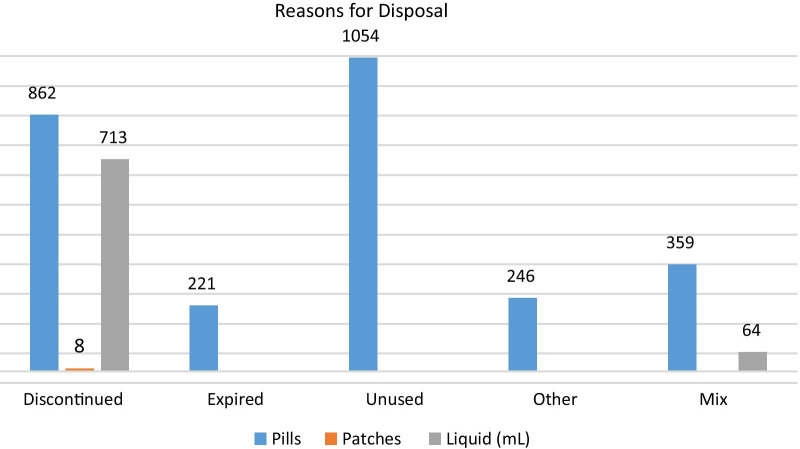
Reasons for medication disposal. Reasons for medication disposal. Participant self-identified reasons for disposing medication which included: discontinued use, expired medication, unused medication, for other reasons, or mixed reasons. Unused medication was the leading reason for disposal

Unused medications were the leading reason for disposal, followed by discontinued medications. Of the 8011 pills destroyed, 1054 were unused; 862 were discontinued; 221 were disposed due to being expired; 359 were a combination of being discontinued, expired, or unused; and, 246 were due to “other” reasons not listed. Of the 777 mL liquids, 713 mL were neutralized due to discontinued use, and 64 mL were disposed of for varied reasons (e.g., discontinued and expired). All eight patches were destroyed due to discontinued use (Fig. 1).

**Fig. 2:**
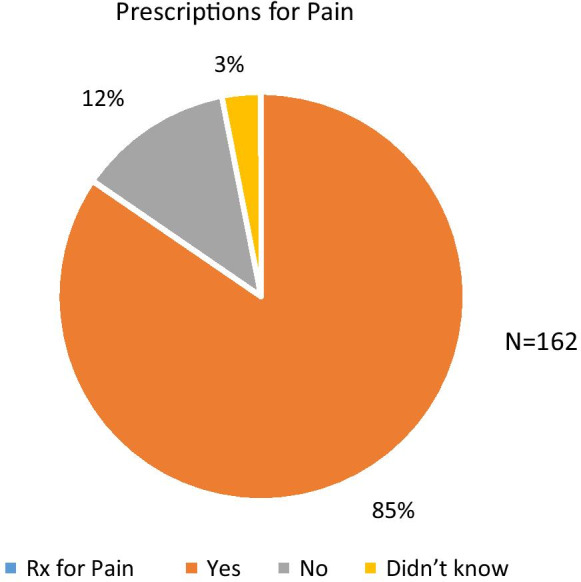
Prescriptions for pain. Prescriptions for pain. Participants self-identified the nature of prescription was pain medication. Majority of the prescription were identified as not for pain

Eighty-five percent (n = 137) of households reported “Yes” that they were disposing of prescription pain medication, while 12% (n = 20) reported “No” to disposing of pain medication; and 3% (n = 5) reported, “Didn't Know" if they were disposing of pain medication (Fig. 2).

**Fig. 3:**
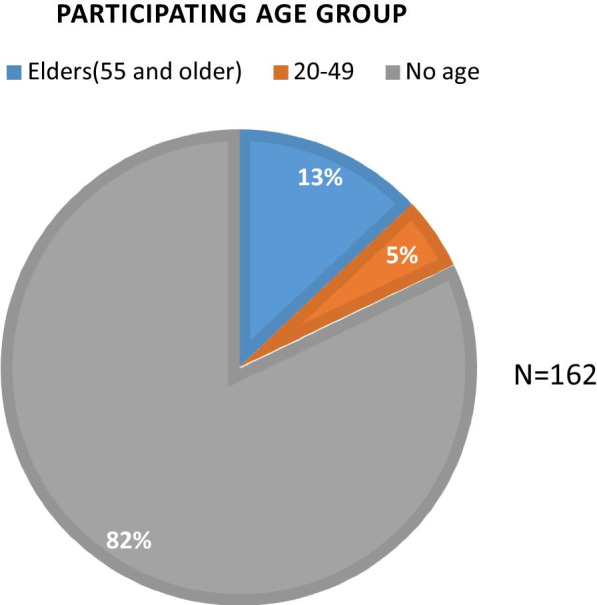
Participants by age. Participants by age. Age of participants that disposed of medication using drug deactivation bags. Elders 55 and older, overwhelming participated in this medication disposal project

Of persons in the participating 162 households, 82% (n = 133) did not self-report their age; 13% (n = 21) identified as elders (i.e., 55 and above age group); and 5% (n = 8) identified as adults (i.e., 20–49 age group) (Fig. 3).

### Medication drop boxes

From October 1, 2018, to September 30, 2020 data were collected from 34 drop boxes located in 29 healthcare facilities. A total of 4684 pounds of medication were collected and disposed of through healthcare facility drop boxes. Ninety percent of the boxes were located in rural settings (Table 2).

**Table 2:** Forms of medications disposed of using the deactivation

Setting	Number of sites	Number of drop boxes	Pounds disposed FY19	Pounds disposed FY20	Total pounds disposed
Rural	26	31	1506.73	2506.05	4012.78
Urban	3	3	297.8	373.45	671.25
Grand total	29	34	1804.53	2879.5	4684.03

Federal healthcare facilities disposed of 3367 pounds of medication during the project period (Table 3).

**Table 3:** Forms of medications disposed of using the deactivation

Site	Number of sites	Number of drop box	Pounds disposed FY19	Pounds disposed FY20	Total pounds disposed
Federal	17	20	1636.43	1730.1	3366.53
Tribal	12	14	168.1	1149.4	1317.5
Grand total	29	34	1804.53	2879.5	4684.03

## Discussion

Based on findings from this project, limited knowledge and access to medication disposal can lead to stockpiling medications in the home, potentially leading to increased diversion, theft or abuse. Many communities in rural settings do not have the same access as those in urban settings. Collection differences in rural communities may also be attributed to lack of access to retail pharmacies or other limitations such as lack of available resources for medication disposal.

The two interventions selected for this project were a result of the community engagement process. This led to increased acceptance and participation. Prior to the project, medication disposal options were limited. At completion of the project, multiple options were available and used. Partnering with healthcare facilities contributed to the success of this project due to their convenience and visitation frequency by the community members. Access to disposal in the home environment was also a convenient alternative for rural tribal communities. This project benefited from the multiple partnerships that exist in tribal communities. The CHR and PHN programs that conduct home health services were critical to the implementation of the drug deactivation bags in the home environment. These programs were instrumental in providing education for proper medication disposal in addition to identifying recipients of resources made available by state partners.

### Limitation

Data collection instruments designed for this project were not end user friendly. As a result, there were gaps in collecting information regarding types of medications disposed. Moreover, to further validate the findings of this project, additional marketing would be needed to expand the number of participants. Lastly, materials used for this project required external funding and future projects would have to identify funding sources. An additional barrier to replicate this project on a larger scale is to identify community partners to distribute and market project interventions.

## Conclusion

The project assessed distribution of drug deactivation bags and medication drop boxes, which proved to be acceptable options for medication disposal in tribal communities. Community education coupled with access to effective disposal options in the home environment and local healthcare facilities resulted in collection of unused/expired medications and reduced diversion risk. The strategies identified in this project are scalable and easy to replicate to address safe medication disposal.

## Data Availability

The datasets used and/or analyzed during the current study are available from the corresponding author on reasonable request.

## Abbreviations

AI/AN: American Indians/Alaska Natives
CHR: Community Health Representatives
DEA: Drug Enforcement Administration
FDA: Food and Drug Administration
FY: Fiscal Year
IHS: Indian Health Services
IPP: Injury Prevention Program
PHN: Public Health Nurses
POC: Point of Contact
